# PrEP Use and Willingness Cascades Among Transgender Women in 11 Asian Countries: Findings From the PrEP APPEAL Cross‐Sectional Online Survey

**DOI:** 10.1002/jia2.70209

**Published:** 2026-10-07

**Authors:** Alegra Wolter, Gede Benny Setia Wirawan, Curtis Chan, Rena Janamnuaysook, Warittha Tieosapjaroen, Hua Boonyapisomparn, Michael M. Cassell, Lei Zhang, Heather‐Marie Ann Schmidt, Jason J. Ong, Nittaya Phanuphak, Benjamin R. Bavinton

**Affiliations:** ^1^ Kirby Institute University of New South Wales (UNSW) Sydney Sydney New South Wales Australia; ^2^ School of Population Health UNSW Sydney Sydney New South Wales Australia; ^3^ Center for Public Health Innovation Faculty of Medicine Udayana University Denpasar Indonesia; ^4^ Institute of HIV Research and Innovation (IHRI) Bangkok Thailand; ^5^ Amsterdam University Medical Centers (UMC) Department of Global Health and Amsterdam Institute for Global Health and Development (AIGHD) University of Amsterdam Amsterdam the Netherlands; ^6^ Amsterdam Institute for Immunology & Infectious Diseases Amsterdam the Netherlands; ^7^ Center of Excellence in Transgender Health Chulalongkorn University Bangkok Thailand; ^8^ School of Translational Medicine Monash University Melbourne Australia; ^9^ Melbourne Sexual Health Centre Alfred Health Melbourne Australia; ^10^ Asia Pacific Transgender Network (APTN) Bangkok Thailand; ^11^ FHI 360 Atlanta Georgia USA; ^12^ Artificial Intelligence and Modelling in Epidemiology Program Melbourne Sexual Health Centre Alfred Health Melbourne Australia; ^13^ China–Australia Joint Research Center for Infectious Diseases School of Public Health Xi'an Jiaotong University Health Science Center Shaanxi China; ^14^ Joint United Nations Programme on HIV/AIDS (UNAIDS) Geneva Switzerland; ^15^ World Health Organization (WHO) Geneva Switzerland

**Keywords:** Asia, HIV cascade, HIV prevention, Pre‐exposure prophylaxis (PrEP), Transgender women

## Abstract

**Introduction:**

Transgender women in Asia face a high HIV burden, yet remain underrepresented in pre‐exposure prophylaxis (PrEP) research. Despite increases in PrEP availability, uptake among trans women remains low. This study examines PrEP awareness, use, and willingness among trans women across 11 Asian countries.

**Methods:**

Data were drawn from the 2022 PrEP Product Awareness, Preferences, and Past Experiences Among Transgender Women and Men Who Have Sex with Men (PrEP APPEAL) study, a multi‐country cross‐sectional online survey. Two PrEP cascades were constructed: one assessing use (awareness, lifetime use, current use) and the other assessing willingness (risk, interest, and willingness to pay). Logistic regression models were used to identify factors associated with lifetime PrEP use and willingness to use PrEP.

**Results:**

Of 1178 trans women participants, 80.0% (*n* = 942) had heard of PrEP. Among those who were PrEP‐aware, 62.6% (*n* = 590/942) reported ever using it, and of these, 78.1% (*n* = 461/590) were current users. Among PrEP‐naïve participants classified as at risk of HIV (*n* = 265), 60.0% (*n* = 159/265) were willing to initiate PrEP, yet only 34.0% (*n* = 54/159) were also willing to pay. Lifetime PrEP use was positively associated with wider country‐level PrEP access (aOR = 4.34; 95% CI: 3.03–6.21), genital surgery (aOR = 1.54; 1.03–2.31), current romantic relationship (aOR = 1.65; 1.19–2.29), as well as reporting sex work (aOR = 1.51; 1.05–2.18), condomless sex (aOR = 1.76; 1.28–2.42), chemsex (aOR = 1.98; 1.36–2.87), and a recent sexually transmitted infection diagnosis (aOR = 2.28; 1.45–3.59) within the past 6 months. Negative associations included older age (aOR per year = 0.94; 0.92–0.96) and discrimination from healthcare workers (aOR = 0.66; 0.47–0.93). Willingness to use PrEP was lower among older participants (aOR per year = 0.97; 0.94–0.99) and those in countries with wider PrEP access (aOR = 0.53; 0.29–0.97), with no significant associations observed for behavioural risks.

**Conclusions:**

PrEP awareness was high among trans women in Asia. PrEP use and willingness were influenced by structural barriers (e.g. country‐level PrEP access), healthcare discrimination, and personal factors (e.g. behavioural risks). Scaling up PrEP for trans women requires rights‐based approaches and commitment to achieve equitable HIV prevention in the region.

## 1 Introduction

Transgender women face a high HIV burden [1]. A 2021 meta‐analysis estimated a global HIV prevalence of 19.9% among trans women [2]. In Asia‐Pacific, HIV prevalence among the trans population was 3.1% between 2020 and 2024, compared to 0.2% in the general population aged 15–49 [3]. Trans women face unique syndemic challenges related to drug use and violence [4, 5]. These vulnerabilities are exacerbated by systemic exclusion [1, 6].

Pre‐exposure prophylaxis (PrEP) is an effective biomedical HIV prevention tool [7, 8, 9]. Joint United Nations Programme on HIV/AIDS set a global target of 21.2 million PrEP users by 2025, prioritising key populations, including trans women. However, by 2024, only 3.9 million individuals worldwide had ever used PrEP [1]. In Asia‐Pacific, PrEP uptake remains low, with fewer than a quarter of a million people using it [3]. Despite PrEP's potential cost‐effectiveness [10, 11], implementation in Asia has been limited, hindered by restrictive policies and shifting sociopolitical climates that negatively impact trans rights [3, 12].

Studies reported that although initial knowledge of PrEP among trans women was low, interest increased once participants were informed [9, 13]. Barriers to PrEP include non‐existent PrEP programmes, limited access, high cost, stigma, discrimination, restrictive laws, and low awareness [14, 15, 16]. Additional challenges include the lack of trans‐specific marketing and prioritisation of hormone therapy over PrEP [9].

PrEP cascades are key tools for tracking implementation but vary by context [17]. In settings with wide PrEP availability, cascades typically assess factors such as eligibility, awareness, uptake, and continuation or adherence [18, 19]. In limited‐access settings, cascades focusing on interest, such as awareness, willingness to undergo clinical procedures, and willingness to pay, can be more appropriate [20]. Despite HIV prevention needs in Asia, data on trans women remain limited [21], and are often conflated with men who have sex with men (MSM) [14, 22].

In 2022, the PrEP Product Awareness, Preferences, and Past Experiences Among Transgender Women and Men Who Have Sex with Men (PrEP APPEAL) study was conducted among trans women and MSM across Asia and Australia [23]. In this paper, we focus exclusively on trans women in Asia to: (1) construct cascades of PrEP use and willingness, and (2) identify factors associated with both outcomes.

## 2 Methods

### 2.1 Study Design, Settings, and Participants

PrEP APPEAL study was a multi‐country, anonymous, cross‐sectional online survey conducted between 10 May and 30 November 2022. Participants were recruited from 11 Asian countries: Cambodia, China (excluding Hong Kong), India, Indonesia, Lao People's Democratic Republic (PDR), Malaysia, Myanmar, Nepal, the Philippines, Thailand, and Vietnam [23].

Participants were eligible if they self‐identified as trans women, 18 or older, self‐reported being HIV‐negative, and lived in a participating country. Recruitment was conducted online via paid and unpaid advertising. Paid advertisements were placed on dating and social media platforms (e.g. Grindr, Facebook). Paid social media influencers promoted the survey, while unpaid community‐based promotion was conducted by regional organisations (Asia Pacific Coalition on Male Sexual Health/APCOM; Asia Pacific Transgender Network/APTN) through their social media, emails, and community networks. An optional prize draw offered one prize per country (3.5% of that country's median income) [23].

Survey instruments were developed in English through multiple rounds of stakeholder consultations, then professionally translated and reviewed by native speakers into primary languages in participating countries: Khmer (Cambodia), Simplified Chinese (China), Bengali, English, Gujarati, Hindi, and Marathi (India), Bahasa Indonesia (Indonesia), Lao (Lao PDR), Bahasa Melayu, English, and Simplified Chinese (Malaysia), Burmese (Myanmar), Nepali (Nepal), English (the Philippines), Thai (Thailand), and Vietnamese (Vietnam). Pilot testing was conducted with target populations in each country, and translations were refined based on feedback [23].

The study was approved by the University of New South Wales (UNSW) Human Research Ethics Committee (HC210729), and subsequently submitted to the World Health Organization Ethics Review Committee (ERC.0003690), which granted an exemption from review as the study met minimal‐risk criteria (anonymous online surveys, no personal data, compliance with the General Data Protection Regulation). Participants provided informed consent electronically. Data were stored on UNSW Sydney's encrypted servers.

### 2.2 Variables

The survey explored sociodemographics, PrEP awareness, use, and preferences, service delivery, attitudes, and risks. Relevant analytic variables were identified a priori by the research team [23]. Aligning with PrEP evaluation indicators, two cascades were constructed: PrEP use (uptake and continuation) and PrEP willingness (demand among non‐users at risk of HIV). This approach mirrors that of Wirawan and colleagues [24], who previously applied it to MSM populations in Asia, and is grounded in the HIV prevention cascade framework proposed by Moorhouse and colleagues [25], with an added step to identify those at elevated HIV risks, and most likely to benefit from PrEP [26].

The PrEP use cascade consisted of three steps: (1) PrEP awareness (“*Have you ever heard of PrEP?*”); (2) lifetime PrEP use (“*Have you ever taken PrEP?*”); and (3) current PrEP use (“*Are you currently taking PrEP?*”). Participants who were PrEP‐aware but had never used it—defined as PrEP‐naïve—were included in a separate cascade assessing willingness to initiate PrEP: (1) at higher risk for HIV; (2) willingness to use PrEP (“*Would you like to take PrEP but have not?*”); and (3) willingness to pay for PrEP [23].

HIV‐related behavioural risk variables were defined as self‐reported behaviours occurring in the past 6 months. Participants were classified as having higher HIV risk if they reported any of the following: more than five sex partners, condomless anal or vaginal sexual intercourse with casual partners, drug use for the purposes of sex (chemsex), injecting drug use (IDU; excluding hormones), history of receiving payments or gifts for sex (paid sex, sex work), and diagnoses of any sexually transmitted infections (STIs) [24, 26]. Willingness to pay was initially assessed using an ordinal scale (“*How much would you be willing to spend per month in total on PrEP, including medication and any costs associated with clinic visits and tests?*”), and was later dichotomised into “willing” or “unwilling.” PrEP‐experienced participants who had discontinued PrEP, as well as PrEP‐naïve individuals who expressed willingness to use PrEP but had not initiated it despite being at risk, were asked to select all applicable reasons for non‐use from a predefined list [23].

Sociodemographic factors included age, education, employment, and residential setting (urbanicity). Countries were classified into two categories based on PrEP integration into national public health systems, determined collectively by the research team using the available programmatic information [23]. Countries with limited PrEP integration (partial access) were those where PrEP was available primarily through pilot trials, post‐trial rollout or otherwise had limited availability (China, India, Indonesia, Lao PDR, Malaysia, Myanmar, Nepal, and the Philippines). Countries with wider PrEP integration (wider access) were those where PrEP had been, or was in the process of being, integrated into the public health system (Cambodia, Thailand, and Vietnam) [23].

Gender affirmation variables included hormone use and genital surgery history. Relationships, sexuality, and social engagement included current romantic relationship status, gender(s) of sexual partners in the past 6 months, HIV testing history, and social engagement with LGBTQ+ friends. Social engagement was assessed using an established two‐item scale (proportion of friends who are LGBTQ+ and amount of free time spent with them) [27, 28]. Responses were scored from 0 to 4 and summed (range: 0–8), then categorised into low (0–2), medium (3–5), or high (6–8) engagement.

PrEP attitude variables included willingness to use PrEP to prevent HIV, concerns about side effects and potential interactions with hormones, comfort discussing PrEP with healthcare providers, preference to discuss PrEP with a transgender healthcare provider, perceived provider awareness of one's gender identity, and experiences of healthcare discrimination. All items were measured on a 5‐point Likert scale and dichotomised into “agree” and “not agree” for analysis.

### 2.3 Statistical Analysis

All analyses were conducted in Stata version 18.0, Basic Edition (StataCorp, College Station, USA). Missing data on any of the four PrEP cascade outcomes (PrEP awareness, lifetime PrEP use, current PrEP use, and willingness to use PrEP) were excluded using a complete‐case approach. For other variables, missing responses were retained and coded in the reference category (0%–8.1% missing).

Descriptive analyses were conducted to summarise study variables. Categorical variables were reported as frequencies and proportions, while age was treated as a continuous variable and summarised using median and interquartile range. Bivariable and multivariable logistic regression models were conducted to assess factors associated with two main outcomes: (1) lifetime PrEP use and (2) willingness to use PrEP. Variables with *p*<0.10 in bivariable models were considered for inclusion in the multivariable models, and then block‐entered, consistent with exploratory epidemiological practice [29]. Odds ratios (OR), adjusted odds ratios (aORs), 95% confidence intervals (CIs), and *p*‐values were reported.

Linearity between age and the log‐odds of each outcome was assessed by modelling age as a four‐level categorical variable and plotting coefficients against group midpoints. Model fit was assessed using the Hosmer–Lemeshow test across 8, 10, and 12 quantiles, with all models demonstrating acceptable fit (*p*>0.05). No convergence issues were encountered [30].

## 3 Results

### 3.1 Participant Characteristics

Of 1260 participants who initiated the survey, 1178 were included after excluding those with missing PrEP cascade outcomes. Table 1 outlines the demographic and participant characteristics. The median age was 26 years (IQR: 23–32). Just under one‐third (30.6%) of participants had completed tertiary education, while 40.9% (*n* = 482) reported being in full‐time employment. About half of participants resided in major cities (*n* = 619, 52.6%), and 57.1% (*n* = 673) were from countries with partial access to PrEP.

**TABLE 1:** Demographic and participant characteristics (*N* = 1178).

Variables	*n* (%)^a^
Age in years, median (IQR)	26 (23–32)
Country of residence	
Cambodia	77 (6.5%)
China (excluding Hong Kong)	24 (2.0%)
India	106 (9.0%)
Indonesia	65 (5.5%)
Lao People's Democratic Republic	25 (2.1%)
Malaysia	10 (0.9%)
Myanmar	115 (9.8%)
Nepal	265 (22.4%)
Philippines	63 (5.4%)
Thailand	207 (17.6%)
Vietnam	221 (18.8%)
Education	
Without high school education	278 (23.6)
High school education	376 (31.9)
Trade/vocational certificate/diploma	164 (13.9)
Undergraduate degree	274 (23.3)
Postgraduate degree	86 (7.3)
Employment	
Full‐time employment	482 (40.9)
Part‐time employment	226 (19.2)
Not working (unemployed, retired)	285 (24.2)
Student	136 (11.5)
Other type of employment	49 (4.2)
Residence	
Capital, major city	619 (52.6)
Small city, town	388 (32.9)
Village, rural area	171 (14.5)
PrEP access by country	
Partial access	673 (57.1)
Wider access	505 (42.9)
Took gender‐affirming hormones	
No	446 (37.9)
Yes	732 (62.1)
Had gender‐affirming genital surgery	
No	882 (74.9)
Yes	296 (25.1)
Currently in a romantic relationship	
No	466 (39.6)
Yes	712 (60.4)
Sexual partners in the last 6 months	
Men only	863 (73.3)
Women only	35 (2.9)
Multiple genders (men, women, trans men, trans women, non‐binary)	280 (23.8)
Last HIV test	
Never tested	79 (6.7)
Tested in the last 12 months	980 (83.2)
Tested over 12 months ago	119 (10.1)
Social engagement with LGBTQ+ friends	
Low engagement, with little time spent	237 (20.1)
Medium engagement, with moderate time spent	543 (46.1)
High engagement, with significant time spent	398 (33.8)
Paid sex in the last 6 months	
No	732 (62.1)
Yes	446 (37.9)
Sex work as the primary income^b^	228 (51.1)
Number of sexual partners in the last 6 months	
Five or fewer	759 (64.4)
More than five	419 (35.6)
Condomless sex with casual partners (anal or vaginal) in the last 6 months	
No	506 (43)
Yes	672 (57)
Chemsex in the last 6 months	
No	823 (69.9)
Yes	355 (30.1)
Injected drug use in the last 6 months	
No	975 (82.8)
Yes	203 (17.2)
STI diagnosis in the last 6 months	
No	959 (81.4)
Yes	219 (18.6)
Willing to take PrEP to prevent HIV	
Not agree	358 (30.4)
Agree	820 (69.6)
Worried about PrEP side effects	
Not agree	563 (47.8)
Agree	615 (52.2)
Concerned about PrEP interacting with hormones	
Not agree	538 (45.7)
Agree	640 (54.3)
Comfortable discussing PrEP with a healthcare provider	
Not agree	425 (36.1)
Agree	753 (63.9)
Prefer to discuss PrEP with a transgender healthcare provider	
Not agree	471 (40)
Agree	707 (60)
Healthcare provider knows that I am transgender	
Not agree	428 (36.3)
Agree	750 (63.7)
Experienced discrimination from healthcare workers due to being transgender	
Not agree	767 (65.1)
Agree	411 (34.9)

Abbreviations: chemsex, chemical sex; HIV, human immunodeficiency virus; IQR, interquartile range; LGBTQ+, lesbian, gay, bisexual, transgender, queer, and other sexual and gender minority identities; PrEP, pre‐exposure prophylaxis; STI, sexually transmitted infection.

^a^ All values are presented as *n* (%) unless otherwise specified. Age is reported as median (IQR).

^b^ Among participants who reported engaging in paid sex in the last 6 months (*n* = 446).

Among participants, 62.1% (*n* = 732) reported ever using gender‐affirming hormones, and 25.1% (*n* = 296) reported having undergone genital surgery. Approximately 60.4% of participants (*n* = 712) were currently in a romantic relationship, and 73.3% (*n* = 863) had only male sexual partners in the past 6 months. A total of 83.2% (*n* = 980) had undergone HIV testing within the past year. Most participants (79.9%, *n* = 941) reported moderate‐to‐high levels of social engagement with LGBTQ+ friends.

Over one‐third of participants (37.9%, *n* = 446) reported engaging in paid sex, of whom 51.1% (*n* = 228/446) identified sex work as their primary source of income. In terms of sexual behaviour, 35.6% participants (*n* = 419) had more than five sexual partners, 57.0% (*n* = 672) reported condomless sex with casual partners, 30.1% (*n* = 355) engaged in chemsex, 17.2% (*n* = 203) had injected drugs, and 18.6% (*n* = 219) had received an STI diagnosis in the past 6 months.

About two‐thirds of participants were willing to take PrEP to prevent HIV (69.6%, *n* = 820) and felt comfortable discussing it with a healthcare provider (63.9%, *n* = 753), with more than half (60.0%, *n* = 707) expressing a preference to engage with a transgender healthcare provider. Most participants believed their healthcare provider knew they were transgender (63.7%, *n* = 750), although about one‐third (34.9%, *n* = 411) reported experiencing discrimination in healthcare settings due to being transgender. Over half of participants were concerned about PrEP side effects (52.2%, *n* = 615) and potential interactions with gender‐affirming hormones (54.3%, *n* = 640).

### 3.2 PrEP Cascades

Among the 1178 participants included in the analysis, 80.0% (*n* = 942/1178) had heard of PrEP. Of those who were PrEP‐aware, 62.6% (*n* = 590/942) reported lifetime use, and 78.1% (*n* = 461/590) were currently taking PrEP. Among the 352 participants who were PrEP‐aware and naïve (29.9% of total participants), 75.3% (*n* = 265/352) were classified as being at higher risk of HIV based on behavioural criteria. Within this higher‐risk subgroup, 60.0% (*n* = 159/265) were willing to use PrEP, and of these, 34.0% (*n* = 54/159) were also willing to pay. Cascades are shown in Figure 1.

**FIGURE 1:**
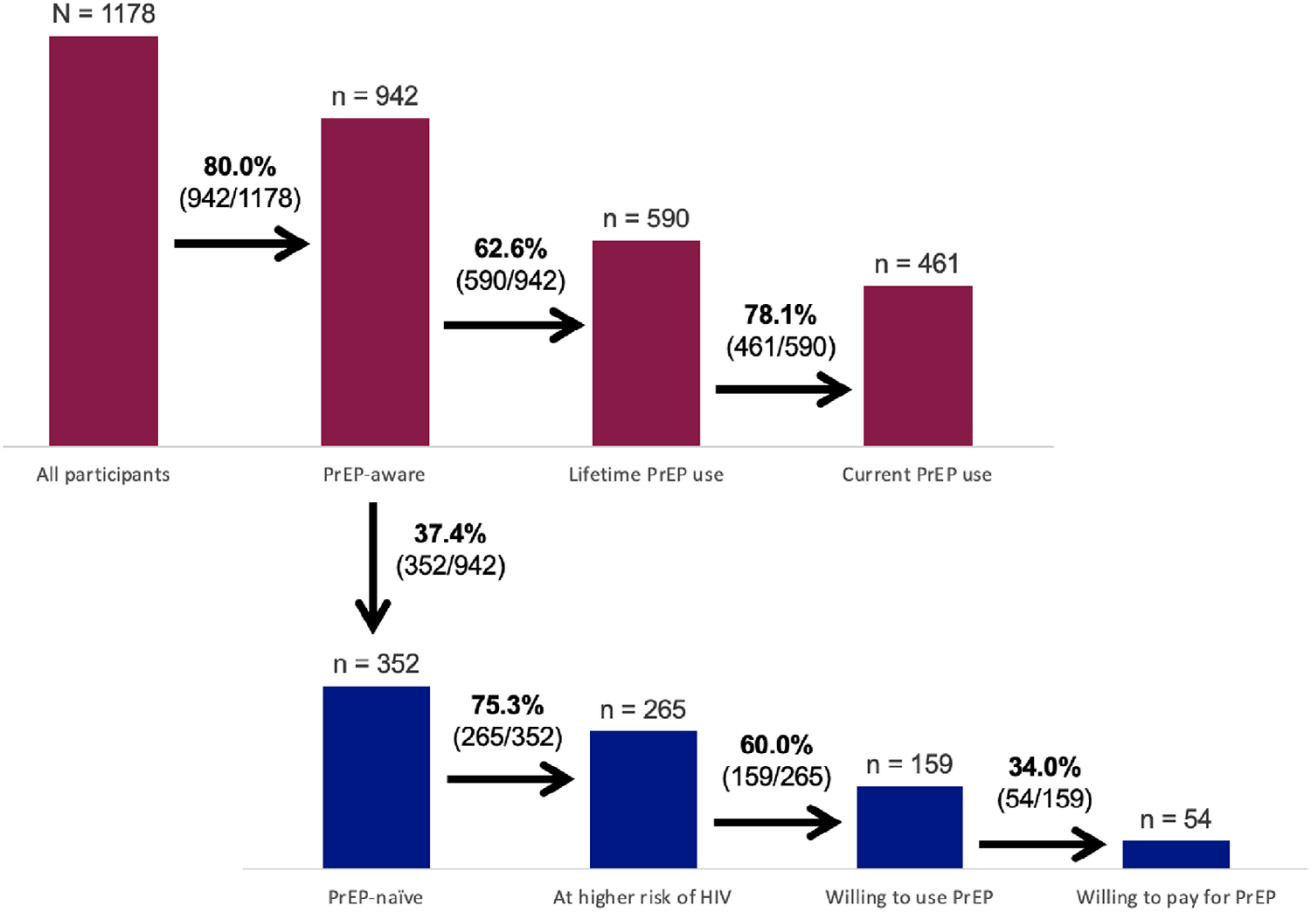
PrEP use and willingness cascades.

When stratified by country‐level PrEP access (Table 2), participants in wider‐access countries reported higher levels of PrEP awareness (85.3%, *n* = 431/505 vs. 75.9%, *n* = 511/673, *p*<0.001), lifetime PrEP use (77.0%, *n* = 332/431 vs. 50.5%, *n* = 258/511, *p*<0.001), compared to those in countries with partial access. Among PrEP‐naïve participants at risk of HIV, willingness to use PrEP was greater in partial‐access countries (63.0% vs. 50.0%, *p* = 0.066), while willingness to pay was lower (30.5% vs. 48.4%, *p* = 0.15). However, neither difference provided strong statistical evidence for a difference between groups.

**TABLE 2:** PrEP use and willingness cascades by country‐level PrEP access.

	Partial access^a^ % (*n*/*N*)	Wider access^b^ % (*n*/*N*)	*p*‐value
PrEP use cascade	*n* = 673	*n* = 505	
PrEP aware	75.9% (511/673)	85.3% (431/505)	<0.001
Lifetime PrEP use	50.5% (258/511)	77.0% (332/431)	<0.001
Current PrEP use	74.8% (193/258)	80.7% (268/332)	0.085
PrEP willingness cascade	*n* = 253	*n* = 99	
At risk of HIV	80.2% (203/253)	62.6% (62/99)	0.001
Willing to use PrEP	63.0% (128/203)	50.0% (31/62)	0.066
Willing to pay for PrEP	30.5% (39/128)	48.4% (15/31)	0.15

*Note*: Denominators are sequential and conditional on the preceding step of the cascade. For instance, “lifetime PrEP use” is calculated among those aware of PrEP.

Abbreviations: HIV, human immunodeficiency virus; PrEP, pre‐exposure prophylaxis.

^a^ Countries with partial PrEP access include China, India, Indonesia, Lao People's Democratic Republic, Malaysia, Myanmar, Nepal and the Philippines.

^b^ Countries with wider PrEP access include Cambodia, Thailand and Vietnam.

### 3.3 Reasons for Discontinuing or Not Initiating PrEP

Reasons for discontinuing or not initiating PrEP are provided in Table 3. Among participants who had taken PrEP but discontinued (*n* = 129), common reasons were personal: 29.5% (*n* = 38) reported not having much sex, 25.6% (*n* = 33) disliked taking pills regularly, and 21.0% (*n* = 27) preferred condoms. Among at‐risk participants who expressed willingness but never initiated PrEP (*n* = 159), access and structural barriers were more prominent: one‐third (32.7%, *n* = 52) did not know where or how to obtain PrEP, 28.3% (*n* = 45) reported PrEP was unavailable where they lived, and 16.4% (*n* = 26) said PrEP was too expensive.

**TABLE 3:** Reasons for discontinuing or not initiating PrEP.

Reasons	Discontinued PrEP use^a^ *n* (%)	Never initiated PrEP^b^ *n* (%)
Personal risk perceptions and preferences		
I am not having much sex	38/129 (29.5)	29/159 (18.2)
I am not at high risk of HIV	17/129 (13.2)	12/159 (7.6)
I am in a monogamous relationship	24/129 (18.6)	9/159 (5.7)
I prefer to use condoms	27/129 (21.0)	27/159 (17)
I do not like taking pills regularly	33/129 (25.6)	45/159 (28.3)
It is too inconvenient for me	11/129 (8.5)	8/159 (5.0)
Health‐related concerns		
I am concerned about side effects	34/129 (26.4)	72/159 (45.3)
Access and structural barriers		
PrEP is not available where I live	16/129 (12.4)	45/159 (28.3)
I do not know where or how to get PrEP	1/129 (0.8)	52/159 (32.7)
I cannot get a prescription for PrEP	6/129 (4.7)	22/159 (13.8)
PrEP is too expensive	6/129 (4.7)	26/159 (16.4)
COVID‐19 made it hard to get PrEP	10/129 (7.8)	15/159 (9.4)
Social and provider‐related barriers		
I was denied access to PrEP by a healthcare provider	0/129 (0.0)	4/159 (2.5)
I do not want to talk about sex with a healthcare provider	3/129 (2.3)	19/159 (12.0)
I am worried about what my friends and family would think	12/129 (9.3)	31/159 (19.5)
My sexual partner(s) do not want me to take PrEP	8/129 (6.2)	4/159 (2.5)

*Note*: Participants could select multiple reasons. Therefore, percentages do not total 100%.

Abbreviations: COVID‐19, coronavirus 2019; HIV, human immunodeficiency virus; PrEP, pre‐exposure prophylaxis.

^a^ Among participants who had ever taken PrEP but were not currently using it (*n* = 129).

^b^ Among interested, at‐risk participants with no prior PrEP use (*n* = 159).

### 3.4 Factors Associated With Lifetime PrEP Use

Bivariable and multivariable logistic regression models were conducted among PrEP‐aware participants (*n* = 942) to identify factors associated with lifetime PrEP use (Table 4). For brevity, only multivariable results with *p* < 0.05 are described in Sections 3.4 and 3.5. Lifetime PrEP use was positively associated with residing in a country with wider access (aOR = 4.34, 95% CI: 3.03–6.21, *p* < 0.001), having undergone genital surgery (aOR = 1.54, 95% CI: 1.03–2.31, *p* = 0.037), being in a romantic relationship (aOR = 1.65, 95% CI: 1.19–2.29, *p* = 0.003), engaging in paid sex (aOR = 1.51, 95% CI: 1.05–2.18, *p* = 0.025), having condomless sex with casual partners (aOR = 1.76, 95% CI: 1.28–2.42, *p* = 0.001), engaging in chemsex (aOR = 1.98, 95% CI: 1.36–2.87, *p* < 0.001), and receiving an STI diagnosis in the past 6 months (aOR = 2.28, 95% CI: 1.45–3.59, *p* < 0.001). Factors negatively associated with PrEP use were: older age (aOR = 0.94 per year increase, 95% CI: 0.92–0.96, *p* < 0.001) and experience of discrimination from healthcare workers due to being transgender (aOR = 0.66, 95% CI: 0.47–0.93, *p* = 0.018).

**TABLE 4:** Logistic regression analyses of factors associated with lifetime PrEP use among PrEP‐aware trans women (*n* = 942).

Variables	PrEP‐experienced, *n* (%)		Bivariable regression		Multivariable regression	
	No (*n* = 352)	Yes (*n* = 590)	OR (95% CI)	*p*‐value	aOR (95% CI)	*p*‐value
Age						
In years, median (IQR)	29 (24–34)	26 (23–30)	0.92 (0.90–0.94)	<0.001	0.94 (0.92–0.96)	<0.001
Education				0.18		
Without university degree	231 (65.6)	412 (69.8)	Ref.			
University degree	121 (34.4)	178 (30.2)	0.82 (0.62–1.09)			
Employment				0.006		0.069
Not employed	100 (28.4)	219 (37.1)	Ref.		Ref.	
Employed	252 (71.6)	371 (62.9)	0.67 (0.51–0.89)		0.72 (0.50–1.03)	
Residence				0.027		0.3
Smaller city, town, village or countryside	138 (39.2)	275 (46.6)	Ref.		Ref.	
Capital or major city	214 (60.8)	315 (53.4)	0.74 (0.56–0.97)		0.82 (0.58–1.15)	
PrEP access by country				<0.001		<0.001
Partial access	253 (71.9)	258 (43.7)	Ref.		Ref.	
Wider access	99 (28.1)	332 (56.3)	3.29 (2.48–4.37)		4.34 (3.03–6.21)	
Took gender‐affirming hormones				0.009		0.9
No	143 (40.6)	190 (32.2)	Ref.		Ref.	
Yes	209 (59.4)	400 (67.8)	1.44 (1.10–1.89)		1.01 (0.72–1.43)	
Had gender‐affirming genital surgery				<0.001		0.037
No	295 (83.8)	394 (66.8)	Ref.		Ref.	
Yes	57 (16.2)	196 (33.2)	2.57 (1.85–3.59)		1.54 (1.03–2.31)	
Currently in a romantic relationship				<0.001		0.003
No	160 (45.4)	184 (31.2)	Ref.		Ref.	
Yes	192 (54.6)	406 (68.8)	1.84 (1.40–2.42)		1.65 (1.19–2.29)	
Social engagement with LGBTQ+ friends				0.3		
Low engagement, with little time spent	57 (16.2)	114 (19.3)	Ref.			
Medium engagement, with moderate time spent	170 (48.3)	254 (43.1)	0.75 (0.51–1.08)			
High engagement, with significant time spent	125 (35.5)	222 (37.6)	0.89 (0.60–1.31)			
Paid sex in the last 6 months				<0.001		0.025
No	234 (66.5)	324 (54.9)	Ref.		Ref.	
Yes	118 (33.5)	266 (45.1)	1.63 (1.24–2.14)		1.51 (1.05–2.18)	
Number of sexual partners in the last 6 months				0.004		0.12
Five or fewer	240 (68.2)	346 (58.6)	Ref.		Ref.	
More than five	112 (31.8)	244 (41.4)	1.51 (1.14–1.99)		1.33 (0.93–1.91)	
Condomless sex with casual partners in the last 6 months				<0.001		0.001
No	184 (52.3)	206 (34.9)	Ref.		Ref.	
Yes	168 (47.7)	384 (65.1)	2.04 (1.56–2.67)		1.76 (1.28–2.42)	
Chemsex in the last 6 months				<0.001		<0.001
No	284 (80.7)	356 (60.3)	Ref.		Ref.	
Yes	68 (19.3)	234 (39.7)	2.75 (2.01–3.75)		1.98 (1.36–2.87)	
Injected drug use in the last 6 months				0.002		0.8
No	301 (85.5)	455 (77.1)	Ref.		Ref.	
Yes	51 (14.5)	135 (22.9)	1.75 (1.23–2.49)		0.93 (0.60–1.47)	
STI diagnosis in the last 6 months				<0.001		<0.001
No	318 (90.3)	426 (72.2)	Ref.		Ref.	
Yes	34 (9.7)	164 (27.8)	3.60 (2.42–5.35)		2.28 (1.45–3.59)	
Worried about PrEP side effects				0.004		0.085
Not agree	149 (42.3)	307 (52)	Ref.		Ref.	
Agree	203 (57.7)	283 (48)	0.68 (0.52–0.88)		0.75 (0.55–1.04)	
Concerned about PrEP interacting with hormones				0.8		
Not agree	167 (47.4)	284 (48.1)	Ref.			
Agree	185 (52.6)	306 (51.9)	0.97 (0.75–1.27)			
Comfortable discussing PrEP with a healthcare provider				0.4		
Not agree	128 (36.4)	199 (33.7)	Ref.			
Agree	224 (63.6)	391 (66.3)	1.12 (0.85–1.48)			
Prefer to discuss PrEP with a transgender healthcare provider				0.4		
Not agree	148 (42)	233 (39.5)	Ref.			
Agree	204 (58)	357 (60.5)	1.11 (0.85–1.45)			
Healthcare provider knows that I am transgender				0.7		
Not agree	121 (34.4)	196 (33.2)	Ref.			
Agree	231 (65.6)	394 (66.8)	1.05 (0.80–1.39)			
Experienced discrimination from healthcare workers due to being transgender				0.058		0.018
Not agree	221 (62.8)	406 (68.8)	Ref.		Ref.	
Agree	131 (37.2)	184 (31.2)	0.76 (0.58–1.01)		0.66 (0.47–0.93)	

Abbreviations: aOR, adjusted odds ratio; chemsex, chemical sex; CI, confidence interval; IQR, interquartile range; LGBTQ+, lesbian, gay, bisexual, transgender, queer, and other sexual and gender minority identities; OR, odds ratio; PrEP, pre‐exposure prophylaxis; STI, sexually transmitted infection.

### 3.5 Factors Associated With PrEP Willingness

Bivariable and multivariable logistic regression models were conducted among at‐risk, PrEP‐naïve participants (*n* = 265) to identify factors associated with PrEP willingness (Table 5). Negative associations were observed for age (aOR = 0.97, 95% CI: 0.94–0.99, *p* = 0.035) and residence in countries with wider PrEP access (aOR = 0.53, 95% CI: 0.29–0.97, *p* = 0.041).

**TABLE 5:** Logistic regression analyses of factors associated with willingness to use PrEP among PrEP‐naïve transgender women reporting HIV‐related risk behaviours (*n* = 265).

Variables	PrEP willingness, *n* (%)		Bivariable regression		Multivariable regression	
	No (*n* = 106)	Yes (*n* = 159)	OR (95% CI)	*p*‐value	aOR (95% CI)	*p*‐value
Age						
In years, median (IQR)	31 (25.5–36.5)	29 (23.5–34.5)	0.96 (0.94–0.99)	0.019	0.97 (0.94–0.99)	0.035
Education				0.075		0.19
Without university degree	70 (66)	121 (76.1)	Ref.		Ref.	
University degree	36 (34)	38 (23.9)	0.61 (0.35–1.05)		0.68 (0.38–1.21)	
Employment				0.3		
Not employed	26 (24.5)	49 (30.8)	Ref.			
Employed	80 (75.5)	110 (69.2)	0.73 (0.42–1.27)			
Residence				0.4		
Smaller city, town, village or countryside	41 (38.7)	70 (44)	Ref.			
Capital or major city	65 (61.3)	89 (56)	0.80 (0.49–1.32)			
PrEP access by country				0.068		0.041
Partial access	75 (70.8)	128 (80.5)	Ref.		Ref.	
Wider access	31 (29.2)	31 (19.5)	0.59 (0.33–1.04)		0.53 (0.29–0.97)	
Took gender‐affirming hormones				0.5		
No	46 (43.4)	63 (39.6)	Ref.			
Yes	60 (56.6)	96 (60.4)	1.17 (0.71–1.92)			
Had gender‐affirming genital surgery				0.9		
No	46 (43.4)	63 (39.6)	Ref.			
Yes	60 (56.6)	96 (60.4)	0.96 (0.49–1.85)			
Currently in a romantic relationship				0.5		
No	45 (42.5)	61 (38.4)	Ref.			
Yes	61 (57.5)	98 (61.6)	1.19 (0.72–1.96)			
Social engagement with LGBTQ+ friends				0.7		
Low engagement, with little time spent	16 (15.1)	26 (16.4)	Ref.			
Medium engagement, with moderate time spent	47 (44.3)	77 (48.4)	1.01 (0.49–2.07)			
High engagement, with significant time spent	43 (40.6)	56 (35.2)	0.80 (0.38–1.68)			
Paid sex in the last 6 months				0.8		
No	58 (54.7)	89 (56)	Ref.			
Yes	48 (45.3)	70 (44)	0.95 (0.58–1.56)			
Number of sexual partners in the last 6 months				0.2		
Five or fewer	66 (62.3)	87 (54.7)	Ref.			
More than five	40 (37.7)	72 (45.3)	1.37 (0.83–2.26)			
Condomless sex with casual partners in the last 6 months				0.6		
No	41 (38.7)	56 (35.2)	Ref.			
Yes	65 (61.3)	103 (64.8)	1.16 (0.70–1.93)			
Chemsex in the last 6 months				0.077		0.3
No	85 (80.2)	112 (70.4)	Ref.		Ref.	
Yes	21 (19.8)	47 (29.6)	1.70 (0.94–3.05)		1.35 (0.73–2.52)	
Injected drug use in the last 6 months				0.088		0.10
No	91 (85.9)	123 (77.4)	Ref.		Ref.	
Yes	15 (14.1)	36 (22.6)	1.78 (0.92–3.44)		1.80 (0.89–3.64)	
STI diagnosis in the last 6 months				0.5		
No	94 (88.7)	137 (86.2)	Ref.			
Yes	12 (11.3)	22 (13.8)	1.26 (0.59–2.67)			
Worried about PrEP side effects				0.18		
Not agree	48 (45.3)	59 (37.1)	Ref.			
Agree	58 (54.7)	100 (62.9)	1.40 (0.85–2.31)			
Concerned about PrEP interacting with hormones				0.3		
Not agree	52 (49.1)	68 (42.8)	Ref.			
Agree	54 (50.9)	91 (57.2)	1.29 (0.79–2.11)			
Comfortable discussing PrEP with a healthcare provider				0.099		0.13
Not agree	34 (32.1)	67 (42.1)	Ref.		Ref.	
Agree	72 (67.9)	92 (57.9)	0.65 (0.39–1.09)		0.66 (0.39–1.13)	
Prefer to discuss PrEP with a transgender healthcare provider				0.7		
Not agree	43 (40.6)	61 (38.4)	Ref.			
Agree	63 (59.4)	98 (61.6)	1.10 (0.66–1.81)			
Healthcare provider knows that I am transgender				0.9		
Not agree	37 (34.9)	54 (34)	Ref.			
Agree	69 (65.1)	105 (66)	1.04 (0.62–1.75)			
Experienced discrimination from healthcare workers due to being transgender				0.4		
Not agree	65 (61.3)	90 (56.6)	Ref.			
Agree	41 (38.7)	69 (43.4)	1.22 (0.74–2.01)			

Abbreviations: aOR, adjusted odds ratio; chemsex, chemical sex; CI, confidence interval; IQR, interquartile range; LGBTQ+, lesbian, gay, bisexual, transgender, queer and other sexual and gender minority identities; OR, odds ratio; PrEP, pre‐exposure prophylaxis; STI, sexually transmitted infection.

## 4 Discussion

To our knowledge, this is the largest multi‐country study of PrEP awareness, uptake, and willingness among trans women in Asia. While 80.0% of the 1178 participants were PrEP‐aware, only 62.6% of those aware had ever used PrEP, and 78.1% of those had remained on PrEP. Among PrEP‐naïve participants, 75.3% were at elevated HIV risk, yet only 60.0% expressed willingness to initiate PrEP—and just 34.0% of them were willing to pay. Multivariable analysis showed that lifetime PrEP use was positively associated with wider country‐level PrEP access, genital surgery, romantic relationship, paid sex, condomless sex, chemsex, and recent STI diagnosis. Negative associations were observed for older age and discrimination from healthcare workers. For PrEP willingness, older age and wider access were negatively associated.

Trans women in countries with wider access to PrEP had over four times the odds of lifetime PrEP use compared to those in partial‐access countries. PrEP awareness, lifetime use, and current use were also higher in these settings (see Table 2). Willingness to initiate PrEP was unexpectedly lower among high‐risk, PrEP‐naïve participants in wider‐access countries, suggesting that country‐level availability alone does not guarantee demand. Factors like distrust [31], perceived stigma, and lack of culturally competent services may undermine healthcare engagement [13, 14, 32]. Although 20 countries in Asia‐Pacific adopted PrEP guidelines in 2023 [33], regional coverage in 2024 remains low, with fewer than 250,000 users, far below the target of 8 million [3]. Such gaps underscore the need for national commitment, including PrEP integration into public health systems [33, 34] and universal health coverage [33, 35, 36]. Given that cost remains a barrier, reflected in low willingness to pay [37], targeted HIV prevention funding in Asia should be considered [38, 39].

About one‐third of participants (34.9%, *n* = 411) experienced discrimination in healthcare settings. Participants who reported discrimination from healthcare workers due to their gender identity had 34% lower odds of using PrEP. This finding reinforces longstanding evidence that stigma hinders access to services [6, 32, 40, 41]. PrEP availability must be paired with non‐discriminatory services [13] that address transphobia [42, 43]. This may include advancing intentional community‐led strategies [40, 44] and tailored health interventions [13, 16, 45]. For example, Thailand's Key Population‐Led Health Services model is a promising differentiated service delivery approach with same‐day PrEP initiation, peer navigation, and community follow‐up, shown to increase uptake and linkage to care [46, 47].

Participants who had undergone gender‐affirming genital surgery had 1.5 times the odds of using PrEP, likely reflecting broader healthcare engagement. Trans women who receive gender‐affirming care tend to have greater socioeconomic stability [48], psychological wellbeing [49], and positive healthcare experiences [9, 50, 51, 52]—all of which may facilitate linkage to services. In this study, hormone use and concerns about drug–hormone interactions were not associated with lifetime PrEP use and willingness to use PrEP. Evidence remains mixed: some studies reported hormone therapy as a barrier due to perceived drug interactions [53] or its prioritisation over PrEP [45, 54], while others found no clear association between hormonal therapy with PrEP uptake [55] or willingness [56]. Relationships between gender affirmation and PrEP use vary by population and context [52]. Our findings challenge the view that drug interaction fears are the main barrier to PrEP uptake, pointing to broader healthcare and structural factors [57]. Importantly, the high proportion of current users among those who had ever taken PrEP (78.1%) suggests that once initiated, many trans women continue use, underscoring the need to facilitate initial uptake [40, 58].

Behavioural risk factors, including condomless sex with casual partners, chemsex, engagement in paid sex, and recent STI diagnoses, were positively associated with lifetime PrEP use. These findings are consistent with previous studies in Asia [34, 59, 60] and globally [61, 62], suggesting that individuals engaging in higher‐risk behaviours are more likely to use PrEP. However, none of the behavioural risk factors were associated with willingness to use PrEP. This mismatch warrants further research and underscores the limitations of biomedical frameworks focused solely on risk eligibility, highlighting the need for person‐centred approaches that consider individual agency [63, 64] and broader contexts in which prevention decisions are made [65].

IDU was not associated with PrEP use or willingness, yet 17.2% of participants reported recent IDU—far exceeding the global estimate of 0.4% among trans people who inject drugs [66]. Additionally, 30.1% reported recent engagement in chemsex, consistent with prevalence ranges in other studies across Asia [67, 68]. Although the high prevalence of IDU and chemsex may partly be due to sampling bias, it underscores the overlooked stigma and criminalisation experienced by trans women in mainstream PrEP delivery [69, 70]. Tailored harm reduction strategies addressing syndemic health disparities are needed [4, 69, 70, 71].

Older age was negatively associated with PrEP use and willingness, consistent with evidence that younger individuals are more likely to engage with PrEP [59, 72]. Being in a romantic relationship was independently associated with higher odds of PrEP use, suggesting that relational dynamics may influence HIV prevention behaviours. One study in the United States found that concerns about a partner's faithfulness led some trans women to view PrEP as necessary, even in monogamous relationships [16]. Exploring how relationships shape perceived HIV risk may guide targeted health promotions. Despite 84.9% of participants reporting medium‐to‐high social engagement with LGBTQ+ peers, this factor was not associated with PrEP use or willingness. While previous studies have linked social relationships to greater PrEP uptake [73, 74], our findings suggest that social connectedness alone may not translate into health‐promoting behaviours, though further research is needed.

Overall, our findings underscore the importance of fostering an enabling environment to support PrEP implementation [14, 33, 75]. Previous studies suggest that PrEP availability alone is insufficient without addressing the underlying structural determinants, such as discrimination, healthcare mistrust, socioeconomic marginalisation and restrictive laws [6, 9, 40, 63]. HIV prevention efforts must centre person‐centred care [9, 63], uphold rights‐based, non‐discriminatory principles [6, 76], ensure universal health coverage [35, 36], and strengthen community‐led service models [4, 43, 44, 77]. Lessons from countries like Argentina, where legal gender recognition and protection are integrated into health systems, highlight potential structural enablers to HIV prevention [57].

This study has several limitations. The cross‐sectional design precludes causal inference. Although this is the largest multi‐country PrEP study among trans women in Asia, the use of a convenience sample may limit generalisability. Given the diversity of participating countries and populations, our findings may not fully capture subcultural nuances, or be representative of all trans women in Asia. Small sample sizes in some countries also precluded country‐specific analyses. HIV and STI status were self‐reported, introducing potential misclassification. Self‐reported measures of sensitive behaviours (e.g. sex work) are subject to recall and social desirability bias. Online recruitment may have excluded individuals with limited digital access, and community‐based sampling may have overrepresented trans women already engaged with HIV services, potentially inflating higher‐risk behaviours (e.g. 17.2% IDU). Regarding IDU, we asked: “*In the last 6 months, have you injected any drugs (excluding hormones)?*” However, the question may have been interpreted differently. Future research should go beyond measuring PrEP uptake to examine systemic barriers and enablers shaping equitable HIV prevention, alongside specific needs of trans women (e.g. gender‐affirming care, relationships).

## 5 Conclusions

There is high PrEP awareness among trans women in Asia. Structural barriers (e.g. access), healthcare discrimination, and personal factors (e.g. behavioural risks) influenced PrEP use and willingness. While country‐level PrEP access was a predictor of use, it did not guarantee demand. Behavioural risks alone were insufficient to explain willingness to use PrEP. PrEP programme scale‐up for trans women requires rights‐based approaches and commitment to achieve equitable HIV prevention in the region.

## Author Contributions

BRB, H‐MAS, JJO, NP and MMC conceptualised and designed the research study. H‐MAS, CC, JJO, MMC, LZ, WT, NP and BRB collected the data. CC conducted the initial data cleaning and was the project manager of the study. AW analysed the data with support from BRB and GBSW, and drafted the manuscript. Writing and analysis were led by trans women researchers (AW, RJ, HB), whose lived experience shaped interpretation, supported by cisgender researchers with technical expertise and long‐term community engagement. All authors reviewed, revised, and approved the final version.

## Funding

This study was supported by funding from the World Health Organization, Kirby Institute, UNSW Sydney, and Outstanding Young Scholars Support Program.

## Conflicts of Interest

BRB received research funding, travel support, and honoraria from ViiV Healthcare and Gilead Sciences. NP received research funding, travel support, and honoraria from ViiV Healthcare and Gilead Sciences (paid to institution). JJO received research funding from ViiV Healthcare and Gilead Sciences (paid to institution). AW, GBSW, CC, RJ, WT, HB, MMC, LZ and H‐MAS have no conflicts of interest.

## Data Availability

A de‐identified copy of the dataset and syntax used in the analysis is available from the authors upon request.
